# Buffalo Academy of Medicine

**Published:** 1901-07

**Authors:** Thomas F. Dwyer


### Society Proceedings.

### BUFFALO ACADEMY OF MEDICINE.

REPORTED BY THOMAS F. DWYER, M. D., Secretary.

ANNUAL meeting held at the Academy rooms, Public Library Building, Tuesday, June 11, 1901. The president, Dr. MARCELL HARTWIG, called the meeting to order at 9 p.m.

The minutes of the last annual meeting were read and approved.

The secretary of the Academy and council presented the following report, for the session of 1900-1901, which, on motion, was received and ordered spread on the minutes:

ANNUAL REPORT OF THE SECRETARY.

*To the Academy of Medicine:*

Twelve applications for membership have been received and elected members during the past fiscal year, of which eleven were resident Fellows and one nonresident Fellow. Twenty-three members, twenty resident and three nonresident, have been dropped for nonpayment of dues, and one resignation has been received and accepted during the year.

The Academy has lost by death one esteemed Fellow, Dr. Frank W. Abbott, who died April 9, 1901.

The Academy was invited by the Medical Society of the County of Erie to participate in a special meeting to take action on Dr. Abbott's death, and at that meeting a committee consisting of Drs. Lucien Howe, De Lancey Rochester and Thomas Lothrop presented an appropriate memorial.

The plan of preparing a program during the summer for the entire session was so successful the past two years, that it was repeated for the session of 1900-1901, by vote of the council, and a copy sent to the members of the Academy.

The constitution and by-laws of the Academy have been revised and a printed copy has been sent to each member.

Only three regular meetings were held and entertained by the sections of the Academy: Section of Obstetrics, June 26, 1900, no quorum; Section of Medicine, September 11, 1900, Clinical quantative estimation of proteid digestion in the stomach, with demonstrations, Dr. A. L. Benedict. Section of Surgery, December 4, 1900, The closure and dressing of abdominal wounds, Dr. Stephen Y. Howell; Treatment of acute general peritonitis, Dr. Eugene A. Smith. Section of Pathology, March 19, 1901, A report of recent investigations of cancer, Dr. H. R. Gaylord; A case of organic epilepsy—clinical and pathological report—Dr. Henry P. Frost.

The meetings of the Academy have been attended as follows: at the September meeting, 21 were present; December meeting, 43; and at the March meeting, 53; an average attendance of 39. I am sorry to report that the attendance has been gradually decreasing for the past two years, not only at the meetings of the Academy, but more particularly at the meetings of the various sections.

The council of the Academy held four meetings, with an average attendance of twelve members.

Respectfully submitted,

THOMAS F. DWYER, Secretary.

The report of the treasurer was read by Dr. Charles S. Jewett. It was moved and seconded that report be received and referred to the trustees as an auditing committee. Carried.

The report of the trustees was read by the chairman, Dr. Benjamin G. Long. On motion the report was received and ordered filed.

Reports were received from the secretaries of the Sections of Medicine, Surgery, Obstetrics, Pathology and Ophthalmology, and were ordered filed.

The tellers, Drs. Blaauw and Borzilleri, reported the result of the election as follows: president, Dr. Charles G. Stockton; secretary, Dr. Francis W. McGuire; treasurer, Dr. Charles S. Jewett; trustee, Dr. Matthew D. Mann.

The retiring president, Dr. Marcell Hartwig, made a few remarks in reference to the work of the past year, and then read his annual address, entitled, Charity by proxy or semi-charity and its disastrous effects upon the physicians. (To be published.)

The auditing committee reported having compared the report of the treasurer with the books and vouchers and found them correct.

The newly elected president, Dr. Charles G. Stockton, was not present, but the secretary, Dr. Francis W. McGuire; treasurer, Dr. Charles S. Jewett, and trustee, Dr. Matthew D. Mann were presented and made brief remarks, thanking the members for having elected them to important offices in the Academy.

Dr. J. W. Grosvenor moved that a vote of thanks be extended to the treasurer for his work during the year. Carried.

Attendance, 32. Adjourned at 10.30 p.m.

---

### *Section on Medicine, May 14, 1901.*

REPORTED BY JULIUS ULLMAN, M.D., Secretary.

THE last regular meeting of the Medical Section of the Buffalo Academy of Medicine for the season was held in the Public Library Building, Tuesday evening, May 14, 1901, with Dr. ELI H. LONG, chairman, presiding.

The order of business being election of officers for the ensuing year, Dr. Julius Ullman and Dr. Albert E. Woehnert were chosen respectively as chairman and secretary by unanimous vote.

Dr. JULIUS ULLMAN reported a case of estivo-autumnal malaria in a patient, aged 32, who had just returned from a long sojourn in Cuba. (See page 892.)

Dr. S. A. KNOPF, of New York, delivered an address on

THE RELATION OF THE MEDICAL PROFESSION IN THE XXTH CENTURY TO THE TUBERCULOSIS PROBLEM.

Dr. Knopf first referred to the energy shown by the medical profession of this age in the treatment of the tuberculosis problem and spoke of the appreciative work of Villemin, who first showed the transmissibility and infectious character of tuberculosis; of Koch for the proofs of the microbic origin, of Cornet, who showed the infectiousness of tuberculous dust; and of Fränkel for his interesting experiments in drop infection. The low death rates from phthisis pulmonalis in the large cities (Paris 4.90, Vienna 3.81, New York 2.26, Berlin 2.19 and London 1.87 per 1,000 inhabitants is to be attributed to the science which the prophylaxis of this disease has reached.

The author alluded to the fallacious idea of the incurability of this disease, which was up to a decade or two entertained by the medical profession, and referred to the thesis of Herman Bremer, who dispelled the idea of its incurability. Bremer introduced the sanitarium treatment of tuberculosis, and not only cured himself, but thousands of others. His successor, Dr. Dittweiler, is also to be remembered for his rest cure therapy in the open air.

America is to be congratulated in not only giving the benefits of the rational treatment of consumption to the well to do, but also to the poor, for the first people's sanitarium for consumption, was begun seventeen years ago, under the direction of Dr. E. L. Trudeau.

The tuberculosis problem must be considered not only in a purely medical, but also in a social aspect. Without statesmen, a liberal municipality and philanthropy, its solution will never be approached, but the medical profession must do the bulk of the work. The physician must not only be a leader but a doctor that is a teacher, and should be well paid for his services. Even the poor should have his family physician, and if unable to pay the physician should be remunerated decently from the public funds. He proved the statement by statistics as to the cost to the state of the maintenance of incurable phthisical patients in the hospitals, as compared to the income such a person could make, provided his disease had been recognised in its incipiency, and undoubtedly cured, so that 75 out of every 100 consumptives would be prevented from becoming a burden to the community.

Under ordinary circumstances everyone is susceptible to tuberculosis, but it is the badly housed, underfed, overworked people, weakened by disease, intemperance and excesses who are the first to fall a prey to the tubercle bacillus; and as physicians we must insist that the dark, dreary, badly ventilated tenement and lodging house should disappear. Especial stress was paid to the underfeeding of the poor school child and some cities were quoted where suitable lunches are given to the child. In this connection the author spoke of the wisdom of the introduction of physicians to examine the children at school.

The author next spoke of the duties of physicians to patients in whom prevention is no longer possible because they are already tuberculous, and advised that every physician should be requested to report to the respective health authorities, the age, profession and residence of every tuberculous individual under his care and only in such cases where the patient or his family wontonly disregard hygienic precaution should the attending physician seek the aid of the health officer to enforce proper hygiene. Treat the consumptive, who is conscientious and does his very best to protect his fellowmen from infection with the utmost kindness, and do not let him feel as if he was an outcast from society.

Dr. Knopf emphasised the importance of educating the masses concerning this disease, congratulating Buffalo on the organisation of an antituberculosis society and urged the great necessity for the formation of a national antituberculosis organisation.

The organisation of a state sanitarium for the treatment of incipient tuberculosis cases in this state is meritorious, but as Da Costa remarked "it seems like a drop of relief in an ocean of woe." There should be a city hospital for consumptives, situated at not too great a distance from the city, and a special city dispensary should be established for the treatment of ambulant tuberculous cases. Besides this there should be a special institution for the treatment of tuberculous and scrofulous children.

Dr. Knopf urged that the physicians and house staff of such institutions should be paid and that every community should have a tuberculosis commission.

DISCUSSION.

Dr. Charles G. Stockton congratulated Dr. Knopf on the intelligent and high-planed presentation of this important subject. He agreed with the doctor on the great importance of nutrition in poor people and said educational means should be adopted to protect ourselves, as well as the community from these cases of phthisis pulmonalis. Following the view of Dr. Knopf, Dr. Stockton favored the establishment of a national association, which will appeal to the laity, and which will educate the masses concerning the nature of the disease and the prophylactic measures to be employed to check its spread. He thought it would be a good move for the Buffalo Academy of Medicine to associate itself with the local antituberculosis society, and take steps toward the formation of a national society.

Dr. John H. Pryor stated that he was heartily in favor of a national antituberculous association, and believed that we should seek the coöperation of New York City physicians, and that an invitation should be extended by the Academy of Medicine of New York to join in the education of the masses on the subject of tuberculosis. Dr. Pryor is of the opinion that other things must be done beside educating. Papers have been published, showing the communicability of the disease, but little has been accomplished. Today the consumptive is abused and neglected. He suffers and gets no relief, because he does not get the proper climatic treatment, and often dies because he is poor. His case is not diagnosticated sufficiently early and because he is tuberculous he succumbs to a second pyemia. Dr. Pryor thought the profession and relatives often indulge in sentiment and that this affects the mortality statistics of this disease. A great many more die of phthisis and other tuberculous diseases than are actually reported. People often falsify the death reports because of industrial insurance. Knowing tuberculosis to be a preventable disease, the state, county and community must provide the means for a man to fight the disease. An excuse is often made because of the large expense which would be incurred, but the loss due to illness and deaths of this disease is sufficient to carry on the entire state government expense. Dr. Pryor advocates state sanatariums and believes each state should follow New York State's example and establish such institutions. Every physician should use his influence on the officials toward this end.

Dr. DeLancey Rochester spoke in favor of the education of the masses and illustrated his remarks, showing the great difficulty recently experienced in Buffalo in having an antispitting ordinance passed by the aldermen. He advocated teaching the school children physiology and hygiene properly, but not from the antiquated and poor text-books now in use. Dr. Rochester thinks that a child is not born tuberculous. Buffalo and Erie County has an institution for the treatment of phthisis pulmonalis, and thought the Erie County Hospital should receive the support which it deserves.

Dr. Benjamin G. Long, president of the Buffalo Antituberculosis Society, advocated the formation of a national society. The society of which he is president, will make an exhibit at the Pan-American Exposition, showing the communicability, preventability and curability of the disease. He also invited all members of the Academy to join the local antituberculosis society.

Dr. Herbert U. Williams thought that the society, as stated above, should stimulate original research in the study of certain problems in tuberculosis. It seems that we have not as yet the whole life history of the tubercle bacillus. The bacillus may exist outside of the body different from those seen in the sputum. The bacillus may resemble somewhat the ray fungus. Bacilli have also been demonstrated in milk and butter, which like the tubercle bacilli, when stained are not decolorised by acid solutions, so that the diagnosis of the tubercle by its characteristic staining properties is not so certain as formerly supposed to be.

Dr. Knopf closed the discussion by saying that climate is not a cure-all, but only an adjuvant in the treatment of phthisis pulmonalis. The education of the masses will be of great service in the future decrease of the mortality of this disease, and for this reason he strongly urges the antituberculosis societies to have lay members.

---

*Section on Ophthalmology, Otology, Rhinology and Laryngology, May 20, 1901.*

Reported by GEORGE F. COTT, M. D., Secretary.

The tenth meeting of this section was held May 20, Dr. F. W. Hinkel in the chair.

Dr. Elmer G. Starr exhibited an instrument for measuring the balance of ocular muscles. Discussion by Drs. Blaauw and Starr.

Dr. John E. Sheppard, of Brooklyn, N.Y., Professor of otology in Long Island Medical College, then read a paper on

SINUS PLEBITIS AND THROMBOSIS.

He said that after a brief historical sketch of the early recognition of sinus disease, a few statistics were presented as to the relative frequency of death from ear disease as compared with the total of deaths, the total of ear diseases, and the total of suppurative ear diseases; as to the relative frequency of the various otitic brain diseases contrasted with one another; and as to the relative frequency of location of otitic thrombosis.

The etiology and pathology were next considered, after which, under symptomatology, were considered seriatim the following: headache, vomiting and constipation, consciousness retained or impaired, restlessness, hyperesthesia, paralyses, vertigo, choked disc and optic neuritis; then the symptoms due to venous obstruction of the sinus, together with those brought about by inflammation and over distension of the superficial veins, viz.: Griesinger's symptom, Gerhardt's symptom, edema of various regions, the hard, tender, cord-like feeling along the anterior margin of the sterno-mastoid muscle, and those due to pressure on the cranial nerves, whose avenue of exit is the jugular foramen, together with those which of all the symptoms are the most characteristic, viz.: the pyemic fever, the chills and rapidly oscillating temperature, and profuse sweating.

Whiting's course of the disease was adopted as being classical. The addition of certain pyemic manifestations to any of the following ear conditions should be considered as diagnostically suggestive: (1) A more or less profuse chronic otorrhea, which has rather suddenly lessened or ceased; (2) a chronic middle-ear suppuration, with such scanty secretion as perhaps to be unknown even to the patient, and discoverable only by ocular inspection under illumination of the external canal and tympanum; (3) an acute middle-ear suppuration attended with more or less unmistakable evidence of mastoid involvement; (4) an acute middle-ear suppuration in which the discharge has gradually lessened and finally ceased from two weeks to two or three months previously, attended with, and followed by, more or less obscure, and slowly lessening symptoms of mastoiditis.

When, therefore, to any of these conditions be added, rigors with rapidly fluctuating temperature, sweating, localised headache, vomiting, especially if associated with any of the evidences of venous obstruction, or the tender, cord-like feeling in the jugular region, and a rapidly developing asthenia, the diagnosis is unmistakable, particularly if there be added thereto metastases. Considerable attention was given to the differential diagnosis, and, under treatment, immediate operation was urged the moment the disease was recognised, the writer also stating that in his opinion it would be a good general rule, to which he admitted a considerable proportion of exceptions, to ligate the internal jugular vein as a measure preliminary to disturbing an infected thrombus in the sinus.

Dr. A. A. Hubbell.—I have had no experience in operations of this condition. I have come across several cases which seemed to be sinus thrombosis. I have seen two cases suspicious of sinus thrombosis and both recovered. We may get complete organisation of clot and get recovery. All patients do not die, even with symptoms of pyemia. In regard to making a diagnosis in cerebellar abscess, I

don't understand what point the doctor has taken in making a diagnosis.

Dr. E. E. Blaauw.—I think we ought to be congratulated for having had the opportunity to listen to Dr. Sheppard's paper. I think the honor of first operating in this condition belongs to Zaufal. He spoke about it in 1888. Operation in meningitis and thrombosis is good. Immense difference whether pneumococci or staphylococci are cause of infection. With the help of the bacteriologist, the burden is lightened. The doctor says there cannot always be made a diagnosis of typhoid fever. There are cases of thrombosis with large spleen, typhoid stools and erythematous patches. We do not blame the general practitioner for making a mistake. The author claims facial vein causes edema of eyelid. That is a mistake. If cavernous vein is affected we get swelling of ophthalmic vein instead.

Dr. Sheppard, in closing, said, a few seemingly authentic cases of sinus diseases have recovered without operation, but it must be remembered that in such cases the diagnosis cannot be confirmed, and at any rate they are so exceptional as to do no more than to prove the rule that the prognosis in unoperated cases is bad. I think perhaps I did not specially emphasise any point in the diagnosis of cerebellar abscess, since that was only casually spoken of as a complicating condition. In this case, and I think that is often true, the possibility of excluding other intracranial conditions aided us as much, perhaps more than, as direct reasoning.

Then followed election of officers: Dr. Elmer G. Starr was elected chairman and Dr. Arthur G. Bennett, secretary.

### REMARKS BY RETIRING CHAIRMAN HINKEL.

In yielding the honors and duties of the chairmanship of this section to my successor. I take pleasure in the thought that in two years of its existence, the section has sanctioned the judgment of the men whose interest and enthusiasm secured its establishment as a separate branch of our Academy of Medicine. It was thought by some that members and interest were lacking to secure the support needful for a department of the Academy devoted exclusively to the consideration of matters pertaining to the eye, ear, nose and throat. The record of the attendance of our meetings of the past year and the character of the transactions, are a sufficient answer to any questions of the advantage to the Academy of the establishment of this section. In members and attendance our meetings have averaged about 30, with a maximum attendance of 87. We have had men of international reputation to address us upon topics pertaining to each of the fields that our section covers, and while technical papers of high quality have not been wanting we have been able also to interest the general profession in our transactions, and one of the best papers of the year was read by a Fellow in general medicine. I believe the section has now passed from the stage of experiment to assured success if we but continue our interest and give the efforts of our officers loyal support. To them it remains to insure the continuance of interest by compelling it by the merit of the themes and speakers presented for our consideration.

Permit me again to urge upon the Fellows the advisability of increasing the clinical features of our transactions. An interesting or obscure case, or one showing the results of operation, or new apparatus or instruments, indeed anything pertaining to the immediate application of the theory of medicine will make our meetings more vital, instructive and profitable, than abstract articles or verbal reports of cases. Let each provide from clinic or private practice, such cases for exhibition as he is able, and I am sure there will be no question of interest in our meetings or of a full attendance. A few of the Fellows have responded well to my request for clinical material and I beg now to thank them for their valuable addition to the work of the year.

By a misunderstanding, the responsibility for which falls upon the chairman, our meetings have been held upon the third Monday of each month. Our by-laws provide that we shall meet upon the second Monday. I suggest that the section do not make the action this year a precedent, but return to the original time. A great advantage to be obtained by holding the meetings on the second Monday of the month, is that it will enable us to hear and adopt the minutes of the previous meeting before they have gone to press. The manuscript for the BUFFALO MEDICAL JOURNAL, in which our transactions appear, must be in the hands of the printer by the 15th of the month, and it happens under our present arrangement that the secretary must have the minutes of the previous meeting in type before they are formally adopted by the section at its succeeding meeting. You will join, I am sure, with the chair in an appreciation of the excellent and full reports of our meetings and discussions that have been made this year by our secretary. In conclusion, I beg to thank the Fellows for the interest they have shown in the work of the section during the year and for the hearty coöperation they have given the officers in their endeavor to make the year's work interesting and worthy.

Before adjournment, Dr. E. E. Blaauw moved a vote of thanks to the essayist, Dr. Sheppard, for his excellent paper, which was unanimously given.

Adjourned.

---

*Section on Pathology, May 21, 1901.*

Reported by JACOB S. OTTO, M. D., Secretary pro tem.

The regular monthly meeting of the Section in Pathology of the Buffalo Academy of Medicine was held in the Academy rooms on the evening of May 21, 1901. Dr. Eugene A. Smith presiding.

In the absence of the secretary, Dr. J. S. Otto was selected pro tem.

The first essayist of the evening was Dr. Herbert U. Williams, professor of pathology, medical department, University of Buffalo, who read a paper on

THE FREQUENCY OF TRICHINOSIS IN MAN IN THE UNITED STATES; ALSO HISTOLOGY OF THE OLD LESIONS.

The microscopic examinations of pork made by the Bureau of Animal Industry have shown that about 2 per cent. of hogs in this country contain trichinæ. Records of autopsies and dissecting rooms in Germany have shown from 0.1 to 3.0 per cent. of cases of trichinosis in man. Osler's records give six cases of trichinosis in 1,000 autopsies. At the Montreal General Hospital trichinosis was found twice in 919 autopsies. It is not certain how far systemic microscopic examinations were carried out in any of the preceding. The writer began investigation seven years ago, and has investigated samples of muscle from 505 unselected autopsies and discovered 27 cases of trichinosis, *i. e.*, 5.34 per cent. The subjects all died of other causes than trichinosis. The trichniæ were invariably encapsulated and often calcified. The infections were of all possible degrees of severity. In some instances the diaphragm was riddled with the parasites, while on one occasion only one, and on another only two trachinæ could be found. The ages of the subjects varied from 19 to 78. The places of birth of the subjects varied as follows:

| | Positive. | Negative. | Total. | Per cent. of + cases in each nationality. |
|---|---|---|---|---|
| United States, white | 6 | 201 | 207 | 2.89 |
| " " negro | 5 | 65 | 70 | 7.14 |
| British and Irish | 5 | 57 | 62 | 8.06 |
| Canadian | 2 | 10 | 12 | 16.66 |
| German | 6 | 43 | 49 | 12.24 |
| Italian | 2 | 10 | 12 | 16.66 |
| Other nationalities | 0 | 27 | 27 | 00.00 |
| Unknown | 1 | 65 | 66 | 1.51 |
| | 27 | 478 | 505 | |

Positive cases were encountered from places as far apart as Buffalo, Baltimore and Denver. An unusually large proportion of cases occurred among insane persons. The histories of the patients seldom made reference to an attack of disease that could have been trichinosis.

Examination of sections of the muscles did not show any collection of eosinophiles or mast-cells about the parasites. Plasma-cells were sometimes found around and within the trichina capsules. Weigert's stain for elastic tissue showed an increase of elastic fibers about the capsules in some cases.

DISCUSSION.

Dr. MATTHEW D. MANN commended Dr. Williams on the good work done and related his experience with trichinosis while a student in the medical college. One case, a man who had eaten raw meat, came to autopsy and many trichniæ were found. Other persons who lived in the locality had the disease and eight cases were diagnosticated. He felt that Dr. Williams's paper ought to arouse painstaking diagnosis.

Dr. ALBERT J. COLTON recalled five cases in the same family which were diagnosticated rheumatism and afterward found to be trichinosis.

Dr. IRVING LYON said that in view of the relation of eosinophilia to trichinosis, it was not surprising that trichniæ had been found in 5 per cent. of cases. He had observed the first case of eosinophilia —that of Dr. Brown at Baltimore, which had 68 per cent. and stated that five cases were diagnosticated thereafter by examination of the blood. Eosinophilia occurs early and persists in some cases four, five or six months. Most frank cases resemble typhoid—some, especially chronic cases, resemble chronic rheumatism. He recalled a case seen recently, that of a Jewish boy, who had eaten raw meat, that had 13 per cent. eosinophiles.

Dr. DeLancey Rochester called attention to one lesson of importance, that of the routine examination of the blood in every acute case of any disease.

Dr. Herbert U. Williams.—Acute cases as a class have not been thoroughly investigated. Is now investigating stomach contents with that in view. Pork is the only meat that is known to be infected. The mouse, fox, squirrel and hedgehog have been found to be infected, but no common food animal other than the pig. Other foods have been examined without result. The rat is the natural host and sausage is the commonest source of infection. The only remedy is the prevention of trichinosis in the hog. The tendon is the most common place of the lesion, but it has been found, though very seldom, in the heart and spleen. It has been demonstrated that migration by way of the peritoneal cavity is not the only way. The young worm may pass through the lymphatics and thoracic duct to the heart and then be carried to any part of the body. Trichinæ have been found in the capillaries.

The second paper of the evening was presented by Dr. Abram T. Kerr, professor of anatomy, Cornell University, the subject being

THE ANATOMY OF THE BRACHIAL PLEXUS.

Dr. Kerr prefaced his paper by stating that many changes were taking place in the subject of gross anatomy, and called particular attention to the phase of embryology by the study of which the questions of the anatomy of the brachial plexus would undoubtedly be solved, for the origin of the plexus can be found by using stains and the microscope. There are a large number of anomalies, and the question arises whether the anomalies are not normal.

It is essential in order to obtain sufficient and accurate statistics that a careful study be made of every subject, and in this connection the speaker urged the training of students in the keeping of complete records of dissection. This plan is carried out by the Anatomical Society of Great Britain and Ireland and the Johns Hopkins University.

It is possible to separate the brachial plexus into types, and in 200 records that the essayist has, the following six types appear :

A—Outer cord from the 4th, 5th, 6th and 7th spinal nerves; posterior cord from the 4th, 5th, 6th, 7th, and 8th spinal nerves; inner cord from the 8th, and 9th spinal nerves, 2½ per cent.

B—Outer cord, 4th and 7th nerves; posterior cord, 4th and 9th nerves; inner cord, 8th and 9th nerves, 43.7 per cent.

C—Outer cord from the 4th and 7th nerves; posterior cord, 4th and 9th nerves; inner cord, 7th, 8th and 9th nerves, 6.2 per cent.

D.—Outer cord, 5th and 7th nerves; posterior cord, 5th and 9th nerves; inner cord, 8th and 9th nerves, 32.1 per cent.

E.—Outer cord, 4th and 5th nerves; posterior cord, 5th and 9th nerves; inner cord, 8th and 9th nerves, 10.5 per cent.

F.—Variable, 5 per cent.

It will be noticed that types B and D together make up 75.8 per cent.

DISCUSSION.

Dr. EUGENE A. SMITH inquired whether any investigations had been made of the anatomy of the external plantar nerve, particularly in relation to Morton's neuralgia.

Dr. KERR stated that he could give no information on this point.

Adjourned, 11 p.m. Present, 28.

---

*Section on Obstetrics and Gynecology, May 28, 1901.*

REPORTED BY WILLIAM G. RING, M. D., Secretary.

The regular monthly meeting was held on Tuesday evening, May 28, 1901, at 8.30 p.m. Dr. HENRY D. INGRAHAM, chairman, pro tem.

The minutes of the previous meeting were read and approved.

Dr. Frank W. McGuire nominated Thomas F. Dwyer for chairman.

Dr. A. L. Benedict moved that the secretary cast a ballot electing Dr. Dwyer. Carried. The secretary so cast the ballot.

Dr. Dwyer made a graceful speech of acceptance.

Dr. Marcel Hartwig nominated Dr. Ludwig Schroeter for secretary. Dr. Charles Jewett nominated Dr. Harry Rooth. Both declined.

Dr. F. W. McGuire moved that a ballot be cast electing Dr. W. H. Mansperger. Carried. The secretary so cast the ballot. Dr. Mansperger accepted in a brief speech.

Dr. A. L. BENEDICT read a paper on the Sanitation, medicine and surgery of the aborigines, with a medical view of their tortures.

DISCUSSION.

Dr. J. W. Grosvenor—I have been greatly interested in this subject. Dr. Benedict has mentioned the objection to trephining. I would like to ask if instruments for performing this operation have been found in the vicinity of the skulls operated on.

Dr. E. E. Blaauw raised a question as to the use of salt among the Indians.

Dr. A. G. Bennett asked if there is any truth that syphilis is an American disease in origin.

Dr. Ludwig Schroeter stated that whole bags of buttons from skulls could be found, showing that trephining was common.

Dr. Benedict, in closing, said methods of drilling were two: by conical instruments and round stalks with sand. We cannot say that syphilis was widespread. There is little tradition of venereal disease. The Indians abhorred salt; they got sufficient from air and meat animals.

Dr. M. D. Mann read a paper on Removal of the female bladder for malignant disease.

DISCUSSION.

Dr. Marcel Hartwig.—Did I understand that Dr. Gaylord found in the urine evidence of the new organism of cancer?

Dr. Grosvenor asked for statistics of recovery.

Dr. Ingraham.—I saw Pawlik's case eleven years ago. The operation of closure of the vagina did not close.

Dr. Mann.—I did not take the percentage of recoveries. I think they are 50 per cent. I have had no trouble in getting perfect closure of vagina.

Dr. Gaylord said the organism that he found was the germ of cancer in abundance in the urine.

Meeting adjourned at 10.45 p.m. Members present, 21.

